# Deep learning can predict cardiovascular events from liver imaging^☆^

**DOI:** 10.1016/j.jhepr.2025.101427

**Published:** 2025-04-22

**Authors:** Gregory Patrick Veldhuizen, Tim Lenz, Didem Cifci, Marko van Treeck, Jan Clusmann, Yazhou Chen, Carolin V. Schneider, Tom Luedde, Peter W. de Leeuw, Ali El-Armouche, Daniel Truhn, Jakob Nikolas Kather

**Affiliations:** 1 Department of Medicine, Section of Hematology/Oncology, The University of Chicago, Chicago, IL, USA; 2 Else Kroener Fresenius Center for Digital Health, Technical University Dresden, Dresden, Germany; 3 Department of Medicine III, University Hospital RWTH Aachen, Aachen, Germany; 4 Clinic for Gastroenterology, Hepatology and Infectious Diseases, University Hospital Duesseldorf, Medical Faculty of Heinrich-Heine-University Dusseldorf, Dusseldorf, Germany; 5 Department of Internal Medicine, Maastricht University Medical Center, Maastricht, The Netherlands; 6 Cardiovascular Research Institute Maastricht (CARIM), Maastricht University, Maastricht The Netherlands; 7 Institute of Pharmacology and Toxicology, Faculty of Medicine Carl Gustav Carus, Technische Universität Dresden, Dresden, Germany; 8 Department of Diagnostic and Interventional Radiology, RWTH Aachen University Aachen, Germany; 9 Department of Medicine I, University Hospital Dresden, Dresden, Germany; 10 Medical Oncology, National Center for Tumor Diseases (NCT), University Hospital Heidelberg, Heidelberg, Germany

**Keywords:** Deep learning, Cardiovascular risk, Liver MRI, Major adverse cardiac events (MACE), Vision transformer (ViT), Self-supervised learning (SSL), UK Biobank, Risk stratification, Biomarker development, Survival analysis

## Abstract

**Background & Aims:**

Cardiovascular mortality remains the leading cause of death and a significant source of morbidity, with metabolic alterations being key etiological factors. As the main metabolic organ, the liver could predict prodromal changes associated with increased cardiovascular risk. However, quantifying this risk remains challenging. This study explores the use of transformer neural networks on liver magnetic resonance imaging (MRI) data to enhance cardiovascular risk prediction.

**Methods:**

Using the extensive collection of liver MRIs in the UK Biobank, we developed a feature extractor with a vision transformer backbone trained in a self-supervised manner. This encoder was then used to predict cardiovascular outcomes from liver MRI scans. Unlike traditional methods, no manual feature selection was required, minimizing bias. Performance was assessed via fivefold cross validation, where predicted risk scores were compared against actual cardiovascular outcomes.

**Results:**

The model was trained on 44,672 liver MRIs. In the fivefold cross-validation predicting major adverse cardiac events, the mean AUC was 0.70 with a 95% CI of 0.69–0.72 and *p* <0.001. The F-statistic from the one-way ANOVA comparing the Systematic Coronary Risk Evaluation 2 (SCORE2) values of the three prediction model score groups was 68.49 with *p* <0.001. The log-rank test comparing the survival of those with prediction model scores above and below 0.5 had a test statistic of 43 and *p* <0.001. The multivariate log-rank test comparing the survival of those in the four quartiles of prediction model scores had a test statistic of 61 and *p* <0.001.

**Conclusions:**

Vision transformer-based models demonstrate promise as quantifiable biomarkers for cardiovascular risk assessment by capturing subtle metabolic and vascular information from liver MRI scans. These findings highlight their strong predictive performance and potential value in risk stratification. Further prospective studies and external validation will be required to establish their clinical utility.

**Impact and implications:**

Our study demonstrates that deep learning applied to liver MRI can predict cardiovascular risk, highlighting the role of the liver as a metabolic indicator of early cardiovascular disease. These findings are significant for clinicians and researchers seeking non-invasive, imaging-based biomarkers for cardiovascular risk stratification, particularly in patients who might not yet exhibit overt symptoms. If validated in prospective studies, this approach could enhance current risk assessment models, allowing for earlier and more personalized interventions in high-risk individuals. However, further validation is necessary before clinical implementation, ensuring broad applicability and integration into existing prevention frameworks.

## Highlights

• Vision transformer from liver MRI predicts major adverse cardiac events (AUC = 0.70).
• Our model rivals established risk scores (*e.g.* SCORE2).
• Hepatic veins and aorta drive major predictive signals.
• Approach requires no manual feature selection or labeling.
• Promising tool for future cardiovascular risk assessment.

## Graphical abstract

**Figure:**
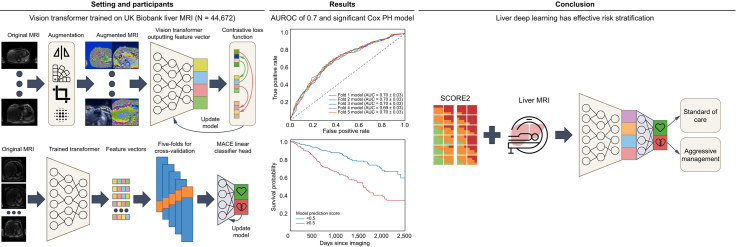

## Introduction

Cardiovascular diseases (CVDs) are the leading cause of morbidity and mortality globally.^1^^,^^2^ Major adverse cardiac events (MACEs), the most clinically relevant form of CVD, stand out as particularly life-threatening and debilitating.^3^ Timely identification of individuals at risk of MACE is vital for improving outcomes and reducing the burden on healthcare systems. Recognizing warning signs and risk factors in advance allows for early interventions, such as lifestyle modifications or medication regimens, which can mitigate the severity or prevent an impending MACE.[4], [5], [6], [7], [8], [9], [10], [11] Furthermore, the economic implications of MACE are high, with substantial healthcare costs associated with hospitalizations, interventions, and long-term care for affected individuals.^12^^,^^13^ In this context, the ability to accurately identify individuals at risk of MACE has emerged as a key area of research and clinical focus. In light of the substantial societal and economic burdens posed by CVDs, the development of new, non-invasive, cost-effective methods for identifying those at elevated risk of MACE is highly relevant.[14], [15], [16], [17], [18]

Cardiovascular morbidity often results from underlying metabolic disorders.[19], [20], [21], [22], [23] The liver is the central metabolic organ, and changes in it, such as increased inflammation or fibrosis, have been shown to pre-date cardiovascular morbidity.^18^^,^[24], [25], [26], [27] However these hepatic changes that precede overt disease are difficult to quantify and do not currently have any significant role in current clinical prediction models for MACE.^28^

The UK Biobank project contains a vast array of health-related data from over half a million participants, including comprehensive medical imaging data, such as single-slice liver magnetic resonance imaging (MRI) scans for a subset of ∼60,000 participants.^29^ These scans, although primarily collected for non-cardiovascular purposes, constitute a largely unused resource for potential insights into cardiovascular health. In addition to health data collected during fixed assessment periods, participant data are additionally updated periodically with information, such as diagnosis and mortality data, through linkage to the National Health Service (NHS) records of all participants. This enables longitudinal analysis of the cohort. This aspect is particularly valuable for studying the progression of cardiovascular risk factors and events, making it an ideal resource for developing predictive models. Furthermore, the UK Biobank dataset encompasses a diverse range of participants from across the British public,^30^ ensuring a representative sample of the population, which is essential for the generalizability of any model developed.

Deep learning (DL) methods make it possible to extract quantitative information from medical imaging data and use it for risk prediction in individual patients.[31], [32], [33], [34] In recent years, the field of medical image analysis has changed substantially following the advent of two new techniques, which further improve the capabilities of DL networks beyond their previous, more limited use cases. The first innovation are transformers, a type of neural network that offers more flexibility compared with previous convolutional neural networks and has been the backbone of many of the latest advances in artificial intelligence (AI) capabilities. The second development is the rapid increase in affordable high-end computation power, which has made self-supervised learning (SSL), a technique to pretrain neural networks without any labels, feasible. The resulting networks of SSL models can be considered as foundation models, which, in turn, can be used to solve supervised downstream tasks more efficiently. Unlike supervised learning, where large amounts of labeled data are required for model training, such foundation models use the inherent structure or content within the data themselves to generate labels or learning tasks.^35^ This is of relevance in the context of medical imaging, where obtaining labeled data for specific tasks, such as predicting MACE, can be costly and time-consuming. By contrast, SSL allows the usage of abundant unlabeled medical image data.

In this study, we trained a liver-MRI foundation model through SSL, specifically applied to single-slice liver MRI images sourced from the UK Biobank dataset. Our primary objective was to predict prior MACE and MACE-related mortality in study participants and subsequently evaluate the predictive ability of the model to forecast future MACE occurrences in participants without a history of MACE-related events before imaging. Our aim was to determine whether liver tissue contains prognostically relevant information for MACE prediction. This could act as a nidus for further investigation into the potential etiological role of liver changes in CVD. On a practical level, we wanted to develop an enhanced biomarker that efficiently incorporates quantitative information about metabolic risk factors, paving the way for more personalized and targeted prevention strategies. The underlying hypothesis of our study posits that single-slice MRI of the liver inherently encapsulates prognostic information, allowing for its extraction to quantitatively predict the risk of future MACE. Using an end-to-end DL approach that foregoes reliance on manually defined features, our model learns directly from liver MRI data in the UK Biobank dataset. Through the integration of SSL into the training process, we aim to quantify liver-related visual information for, and thereby derive accurate predictions of cardiovascular morbidity and mortality, contributing to the development of an effective tool for personalized cardiovascular risk assessment.

## Patients and methods

### Ethics statement

The overall analysis was approved by the Ethics Board at the University Hospital Carl Gustav Carus, Dresden, Germany. This study adhered to the tenets of the Declaration of Helsinki. Written informed consent was obtained from participants in the UK Biobank project.

### Cohort description

The UK Biobank cohort comprises 502,309 individuals aged 40 years and above, residing in the UK, who were recruited for this prospective study between 2006 and 2010. Approximately 5 years after the initial UK Biobank assessments, specifically between 2014 and 2017, 45,422 participants underwent comprehensive MRI body scans, which included the evaluation of abdominal composition. These MRI scans were conducted using Advanced MR Analytics AB (AMRA Medical, Linköping, Sweden).

In the liver MRI acquisition protocol of the UK Biobank, a single transverse slice at the level of the porta hepatis was used to visualize the liver. To obtain the required data, two distinct sequences were used. An initial sequence involved a single breath-hold cardiac-gated T1-mapping Modified Look-Locker Inversion Recovery (T1-MOLLI) sequence, lasting ∼12 s. This sequence yielded ∼10 images per patient following a standardized protocol (6-mm slice thickness, 25-mm in-plane resolution according to the UK Biobank Consortium). The participants used for the present investigation were imaged over an ∼6.5-year-long period from mid-2013 until early 2020. The median imaging date was August 23, 2017 with an SD of 589 days. Follow-up continued from the date of imaging until MACE, death, or September 15, 2021. The mean follow-up time was 1,250 days with an SD of 648 days.

The use of the single-slice T1-MOLLI imaging was not because of a specific selective preference but rather a constraint imposed by the availability of these data within the UK Biobank cohort. The UK Biobank provided only this imaging protocol, which limited our choice in selecting more comprehensive imaging sequences.

International Classification of Diseases, 10th Revision (ICD-10) codes for MACE and death outcomes were obtained through the linkage of participants' records to real-world, administrative data sources, including hospital inpatient and death records and primary care records, as part of the participant information contained within the UK Biobank dataset. MACE was defined in line with the definition used in Systematic Coronary Risk Evaluation 2 (SCORE2) (Table S1).

### Experimental design

#### Cross-validation

To assess the predictive performance of our model, we used a fivefold cross-validation approach. The 44,672 participants used for the training process included a combination of all those with a recorded occurrence of MACE before the liver MRI acquisition date (n = 974), as well as the majority of participants with no history of MACE before image acquisition (n = 43,698 participants). A further 750 participants (all 214 participants with first-time MACE after imaging and 536 randomly selected participants with no history of MACE both before and after imaging) were held out for external validation. Because of changes in local data access policies by the UK Biobank, we did not have access to the UK Biobank liver fat measurement data. In our hold-out cohort, we excluded any participants with known liver disease (*i.e.* a recorded ICD-10 code starting with K70–K77) to ensure that our model findings were largely independent of overt liver disease. The selection of 750 participants for the hold-out cohort was based on statistical power calculations to ensure sufficient robustness in external validation. A power analysis for the receiver operating characteristic (ROC) AUC metric indicated that this sample size provided adequate power (≥95%) to detect a meaningful difference in predictive performance. In addition, survival analysis power calculations demonstrated that the observed 214 MACE events provided sufficient statistical power to detect a hazard ratio of 1.8, ensuring reliable Kaplan–Meier survival estimates. These calculations justify the use of 750 participants as an appropriate external validation set to minimize overfitting while ensuring adequate event representation.

The dataset of first T1-MOLLI sequence images per patient was randomly partitioned into five equal-sized subsets, with each subset serving as the test set exactly once. The remaining four subsets were pooled together to form the training set for model development. This process was repeated five times to ensure that each subset had an opportunity to act as the test set. During each fold of cross-validation, the primary evaluation metric used was the AUC, which quantifies the ability of the model to discriminate between individuals who experienced MACE or MACE-related mortality and those who did not. To mitigate the potential for overfitting, the mean prediction score of all five models per participant was used for survival analyses, with comparison made with the European Society of Cardiology (ESC) SCORE2 model and its subgroup variants (SCORE2-Diabetes and SCORE2-OP),[36], [37], [38] primarily through covariate analyses.

#### Survival analysis

To assess the predictive utility of our model, Kaplan–Meier curve-based survival analysis was performed on a hold-out cohort of randomly selected participants with available liver imaging data and no history of MACE before imagining. Survival outcomes in the form of MACE or MACE-related mortality were compared based on the risk scores generated by mean model prediction score. The log-rank test was used to evaluate the statistical significance of survival differences between participants when dividing them based on median score and tertiles based on their scores.

#### Comparison of liver model and SCORE2

To contextualize the performance of our model, we included participants with both liver imaging data and the required tabular data for the SCORE2, SCORE2-Diabetes, and SCORE2-OP risk models. We compared the distribution of SCORE2 values after dividing the test population into three groups on the basis of each participant's mean model score (<0.33, 0.33–<0.66, and ≥0.66) to provide a general overview of the underlying structure and pattern of the dataset. In addition, we utilized Kaplan–Meier survival analysis to assess survival differences between clinical risk groupings derived from SCORE2. The pairwise log-rank test was used to determine the statistical significance of survival disparities between these groups.

To further evaluate the diagnostic performance of our model, we compared its ROC curve with that of the SCORE2 risk estimate. ROC analysis was used to assess the ability of the models to discriminate between individuals who experienced MACE or MACE-related death and those who did not. The primary metric of interest was the AUC, and statistical comparison between the two ROC curves was conducted using the DeLong test.

In addition to evaluating the predictive performance of our model, we explored the influence of various covariates on the risk of experiencing MACE or death. Hazard ratios (HRs), accompanied by 95% CIs and *p* values, were calculated for multiple covariates, including the risk score of our model, age, smoking status, systolic blood pressure, total cholesterol level, HDL cholesterol level, antihypertensive medication use, and diabetes diagnosis.

We performed a subgroup analysis by stratifying individuals with SCORE2 risk estimates of 5%–<10% into high and low-risk groups based on the model score of the split at ≥0.5. This analysis aimed to assess the ability of our model to differentiate risk within a subset of individuals with uncertain benefit from interventional primary prevention strategies against cardiovascular events, such as initiation of statin-based therapy.^39^

Finally, we compared the distribution of participant subgroups on the basis of identified risk factors from SCORE2 (diabetes status, total cholesterol, HDL cholesterol, systolic blood pressure, sex, and smoking status) within the prediction scores of our model to provide insight into which cardiovascular risk factors were being captured in a more pronounced way by our model.

#### Deep learning models

We used Momentum Contrastive Learning (MoCo) v3^40^ (Siemens 1.5 Tesla MAGNETOM Aera scanner (Siemens Healthineers, Erlangen, Germany)) as the SSL framework to train a vision transformer (ViT)^41^ on the single-sliced liver MRI of the UK Biobank. To use the highest available contrast, we chose the first image of the T1-MOLLI sequence^29^ for each patient. MoCo is a contrastive learning framework that optimizes feature representations of arbitrary structured data by encouraging similarity between corresponding data pairs (stochastically augmented views of the same sample) and dissimilarity between all other stochastically augmented views.

Our model architecture comprised a base ViT (ViT-B) with a convolutional base, as implemented in the official MoCo repository. We pretrained the encoder using contrastive SSL, with hyperparameters set to the default values recommended in the MoCo repository, except for the learning rate, which was reduced to 1 × 10^-4^ for improved training stability, and the batch size, which was set to 1,024 because of hardware limitations. A detailed list of hyperparameters can be found in Table S2 and S3.

After pretraining the encoder, we froze its weights and used it to extract compressed feature vectors from the liver MRI images. These feature vectors served as inputs for a linear classifier with one dense layer, which was trained in a supervised manner on the MACE target.

**Fig. 1:**
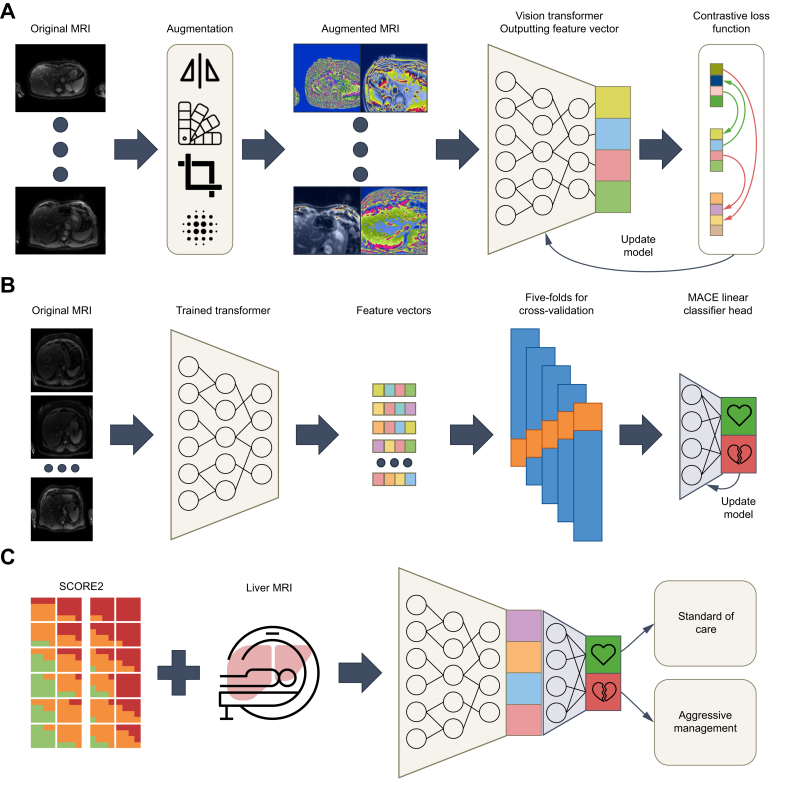
Deep learning overview to predict MACE directly from liver MRI. (A) Illustration of the pretraining using contrastive learning. Various stochastic augmentations are applied to the liver MRI to create multiple views of the same MRI before being processed by the ViT-B, which is trained with a contrastive loss function. (B) The pretrained ViT-B model is harnessed to generate the feature vectors of the MRI, which serve as inputs to the linear classifier head, which is directly trained on the MACE target in a supervised manner. The weights of the feature extractor are frozen for the supervised training. (C) Proposed approach to applying such a model in clinical practice. Should the model determine that the patient is in a low-risk group, management can follow SCORE2-based guidance. However, in the event of the model identifying a high-risk patient, more aggressive management should be used. MACE, major adverse cardiac events; MRI, magnetic resonance imaging; SCORE2, Systematic Coronary Risk Evaluation 2; ViT, vision transformer; ViT-B, base Vit.

To generate heatmaps, we used a technique that combines the gradients of our DL model with the original MRI image pixel values. Specifically, we computed the gradients of the output of the DL model with respect to the input image using backpropagation, which highlights the pixels that most impact the predictions of the model. We then multiplied these gradients element-wise with the original image pixel values, producing a map that represents the importance of each pixel for the decision-making process of the model (Fig. 1).

The resulting heatmaps provide a local approximation of the behavior of the DL model for each input image, allowing us to visualize and understand which regions of the image are most relevant for the predictions. This technique helped us to interpret the model results, identify potential biases, and ensure the reliability of our DL model in medical imaging applications.

#### Code availability

The code used to train the DL models can be found on at https://github.com/KatherLab/liverMRI.

## Results

### Model identifies participants with impending MACE with meaningful substratification in SCORE2 risk categories

**Fig. 2:**
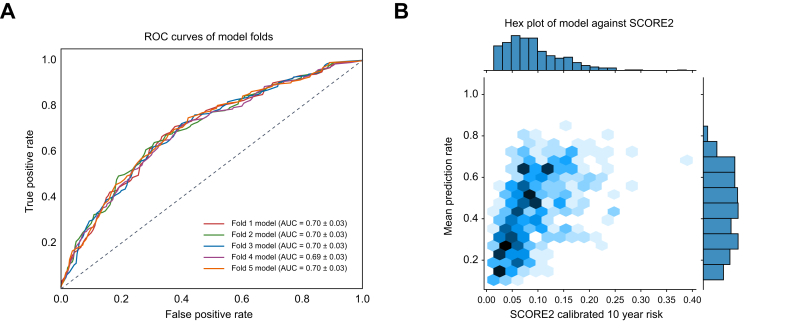
Model cross-validation achieves adequate performance and stratifies broadly in line with SCORE2. (A) ROC curves for the five models created as part of the fivefold cross-validation process. (B) Density estimate plot demonstrating how model prediction scores are distributed relative to the SCORE2 risk estimate in the external validation cohort. ROC, receiver operating characteristic; SCORE2, Systematic Coronary Risk Evaluation 2.

The summarized participant characteristics are detailed in Table S4. We performed fivefold cross-validation on the dataset and achieved a mean AUC of 0.70 (95% CI: 0.69–0.72, *p* <0.001) (Fig. 2A). Given the similar performance and shape of the ROC curves for all five -folds and in the interest of reducing overfitting in any subsequent analyses, we used the intraparticipant mean prediction model (PM) score across all five -folds. The distribution of the scores generated by the prediction model was examined insofar as how they related to the already established SCORE2 model; the Pearson r value was 0.52 with *p* <0.001 (Fig. 2B). We performed a one-way ANOVA, which revealed a statistically significant difference between the three groups (F-statistic 68.49, *p* <0.001). Subsequent pairwise independent *t*-tests with Bonferroni’s correction between all three groups revealed strongly significant stratification between all group pairings (Table 1). These findings demonstrate that the PM model has sufficient discriminatory power to stratify patients based on risk.

**Table 1:** Pairwise log-rank tests for the prediction score groupings on the basis of prediction model distributions demonstrate significant differences between all possible permutations.

Comparison		T-statistic	*p* value
<0.25	0.25–<0.5	1.200	<0.001
	0.5–<0.75	18.895	<0.001
	≥0.75	16.167	<0.001
0.25–<0.5	0.5–<0.75	24.460	<0.001
	≥0.75	2.868	<0.001
0.5–<0.75	≥0.75	10.585	<0.001

**Log-rank tests for SCORE2 groups split at prediction model score <0.5 and ≥0.5**			
**SCORE2 category**		**T-statistic**	***p* value**
<5%		0.752	0.386
5–<10%		2.792	0.095
≥10%		13.198	<0.001

SCORE2, Systematic Coronary Risk Evaluation 2.

### Model score stratifies participants’ long-term risk of MACE and cardiovascular mortality

**Fig. 3:**
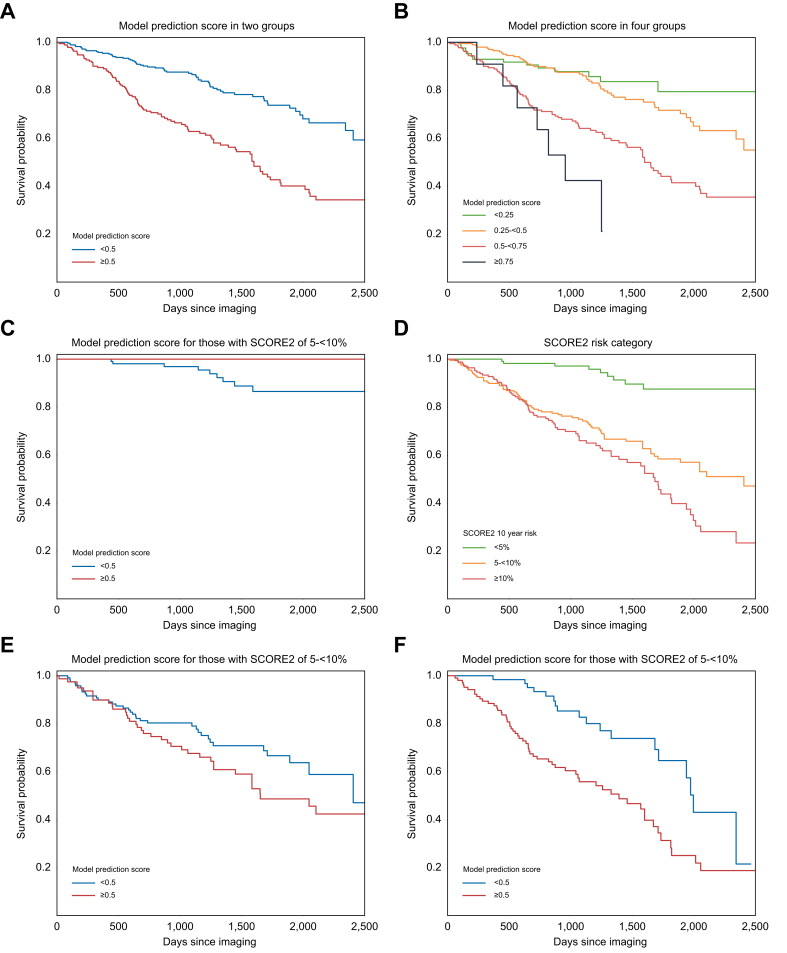
Kaplan–Meier curves from the prediction model scores. Kaplan–Meier curves for participants of the hold-out cohort divided into (A) two groups, those with a MACE prediction score ≥0.5 and those <0.5, where events were MACE and/or cardiovascular-related death and (B) quarters based on their prediction model score, where events were MACE and/or cardiovascular-related death. Kaplan–Meier curves for (C) the subgroup of the hold-out cohort with SCORE2 risk scores of <5% divided into two groups, those with a MACE prediction score ≥0.5 and those <0.5, where events were MACE and/or cardiovascular-related death; (D) participants of the hold-out cohort divided into the three commonly used risk groups when using the SCORE2 model: those with a 10-year risk of a MACE or cardiovascular-related death of <5%, 5–<10%, or ≥10%; (E) the subgroup of the hold-out cohort with SCORE2 risk scores of 5–<10% divided into two groups, those with a MACE prediction score ≥0.5 and those <0.5, where events were MACE and/or cardiovascular-related death; and (F) the subgroup of the hold-out cohort with SCORE2 risk scores of ≥10% divided into two groups, those with a MACE prediction score ≥0.5 and those <0.5, where events were MACE and/or cardiovascular-related death. MACE, major adverse cardiac events; SCORE2, Systematic Coronary Risk Evaluation 2.

We assessed the survival probabilities of all participants with liver imaging who did not have a history of MACE before the imaging investigation being performed but who subsequently had a MACE (*n* = 157) as well as the randomly selected control group who never had a MACE occur (*n* = 320) over the course of the longest possible follow-up period of 2,100 days, with the population split at a prediction score of ≥0.5 (Fig. 3A). Log-rank test analysis revealed a statistically significant difference in survival between the groups (log-rank test 43, *p* <0.001), indicating that our PM score exerts a profound predictive ability on the cardiovascular-related outcomes of our study population.

When the groupings were based on quarters of the PM score, the Kaplan–Meier curves continued to provide a clear stratification of MACE or cardiovascular-related death in the study population (Fig. 3B). This appeared to create a similar stratification to the SCORE2 values (Fig. 3D). Similar to the previous analysis, the curve exhibited noticeable distinctions in the MACE or cardiovascular-related survival trajectories of these three groups with a significant multivariate log-rank test (test statistic = 61, *p* <0.001), indicating that the PM score is a significant factor influencing the overall survival of the study population. The pairwise log-rank test analysis reaffirmed the statistical significance of these differences in survival across all pairings (Table 1). The data showed that the PM score has a major role in predicting survival outcomes in participants with liver imaging who did not have a history of MACE before the imaging investigation, with equivalent stratification to that of SCORE2.

We subsequently sought to compare the predictive performance of the PM relative to a widely used clinically validated metric in the form of the SCORE2 and its derivatives in the form of SCORE2-Diabetes and SCORE2-OP. Not all participants with liver imaging data had complete tabular data necessary to estimate a SCORE2 risk assessment; thus, only participants with both liver imaging data and the relevant tabular data for SCORE2 calculations were included in the following analyses (n = 477).

For comparison, we performed a similar stratification using the well-established SCORE2 cut-offs of <5%, 5–10% and ≥10% risk over a 10-year period. This rendered a Kaplan–Meier curve with similar discriminatory ability to that of the PM. This implies that the PM score, derived from liver imaging data, can provide insights into cardiovascular risk that are comparable to established clinical tools, potentially offering an additional avenue for risk assessment and personalized patient care.

### Model score outperforms SCORE2 in predicting MACE and cardiovascular-related death

**Fig. 4:**
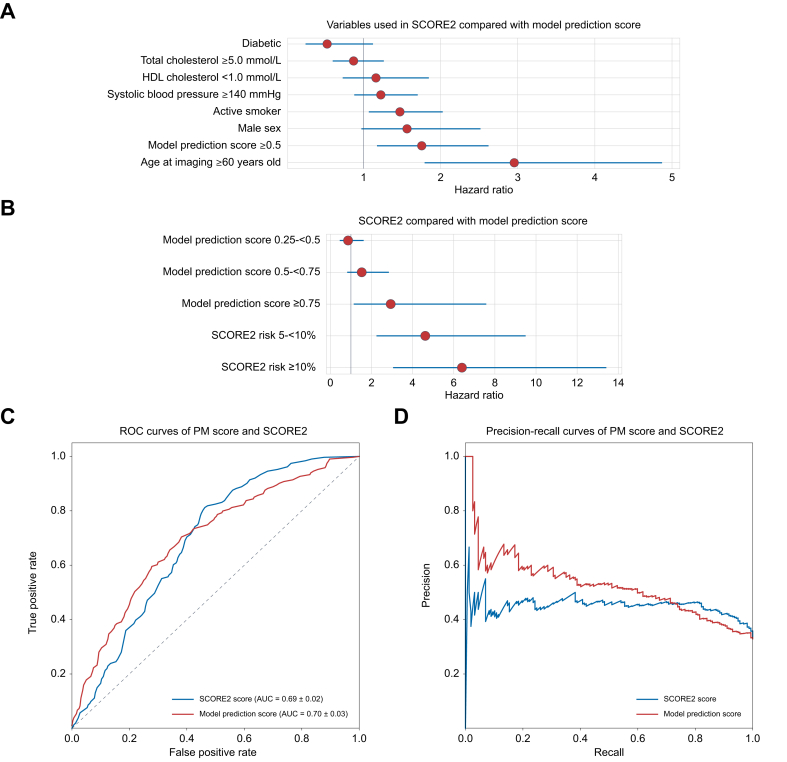
Comparison of the performance of the prediction model with that of SCORE2 in the external validation cohort. (A) Forest plots of the hazard ratios for MACE or cardiovascular-related death from a multivariate Cox proportional hazards regression model of (A) the individual components of SCORE2 score and PM scores and (B) SCORE2 and PM scores. (C) ROC curves directly comparing the predictive performance of the SCORE2 and PM scores. (D) Precision-recall curves of SCORE2 and PM scores. MACE, major adverse cardiac events; PM, prediction model; SCORE2, Systematic Coronary Risk Evaluation 2.

We performed two multivariate Cox proportional hazards regression models: one comparing the PM scores in four groupings split at 0.25, 0.5, and 0.75, with the underlying variables used in SCORE2, and another comparing the PM score in four groupings with SCORE2 directly. When comparing the PM with the underlying variables from SCORE2, the groupings displayed a stepwise increase in their hazard ratios (Table 2, Fig. 4A). This trend continued in the Cox proportional hazards regression model comparing the model with SCORE2 directly with the highest risk group in the PM, which had a HR of 2.934 compared with the SCORE2 ≥10% risk group with a HR of 6.398 (Table 2, Fig. 4B).

**Table 2:** Cox proportional hazard regression model for predicting cardiovascular events, comparing the prediction model to established risk factors in the SCORE2 Model.

Covariate (n = 477)	Hazard ratio	Lower 95% CI	Upper 95% CI	*p* value
Age at imaging ≥60-years old	2.954	1.792	4.870	<0.001∗
Model prediction score ≥0.5	1.754	1.174	2.621	0.006∗
Male sex	1.564	0.972	2.516	0.065
Active smoker	1.472	1.068	2.027	0.018∗
Systolic blood pressure ≥140 mmHg	1.224	0.880	1.703	0.230
HDL cholesterol <1.0 mmol/L	1.161	0.730	1.846	0.528
Total cholesterol ≥5.0 mmol/L	0.871	0.601	1.263	0.466
Diabetes	0.528	0.248	1.123	0.097

**Prediction model compared with SCORE2 risk groups**				
SCORE2 risk ≥10%	6.398	3.051	13.418	<0.001∗
SCORE2 risk 5–<10%	4.615	2.243	9.496	<0.001∗
Model prediction score ≥0.75	2.934	1.135	7.582	0.026∗
Model prediction score 0.5–<0.75	1.525	0.818	2.844	0.184
Model prediction score 0.25–<0.5	0.860	0.457	1.618	0.640

This table presents the hazard ratios and 95% CI for various predictor variables, including age, sex, smoking status, blood pressure, cholesterol levels, and diabetes status. Notably, the prediction score of the model (≥0.5) is significantly and independently associated with an increased risk of cardiovascular events. In addition, age ≥60 years and active smoking are also significant predictors of increased risk. ∗Statistically significant HR with *p* <0.05. SCORE2, Systematic Coronary Risk Evaluation 2.

The AUC and precision recall of the PM and SCORE2 were compared to assess their respective diagnostic performance in distinguishing between those who would experience MACE or cardiovascular death and those who would not. The PM exhibited a higher AUC value of 0.70 (95% CI: 0.67–0.73) compared with SCORE2, which had an AUC of 0.69 (95% CI: 0.67–0.71) (Fig. 4C). When performing the DeLong test, this resulted in a statistically insignificant performance by the PM over SCORE2 (DeLong test statistic = -0.873, *p* = 0.382). The ROC curves visually demonstrated that the PM initially maintained a steeper ascent, reflecting a potentially superior sensitivity across lower specificity levels. In addition, the precision-recall curve of the PM outperformed that of SCORE2, especially at lower recall levels (Fig. 4D).

Finally, we sought to identify whether the PM could meaningfully stratify low and high-risk populations within the various SCORE2 risk groups, thus determining the ability of our model to identify members of the population who require more aggressive preventative measures than current risk assessment measures would suggest. When stratifying the already established SCORE2 risk groups of <5%, 5–<10%, and >10% on the basis of a PM score either <0.5 or ≥0.5, a clear stratification emerged in all groups, with the >10% group being statistically significant with a T-statistic of 13.198 and *p* <0.001 (Table 1, Fig. 3C,3E,F).

### Model score associations with clinical and lifestyle factors independent of SCORE2

We additionally examined how PM scores related to key clinical and lifestyle factors not considered in SCORE2, namely alcohol levels and BMI category. These analyses were conducted separately from the multivariate analysis, because BMI and alcohol consumption are not included in SCORE2. A one-way ANOVA comparing prediction model scores across different BMI categories (normal, overweight, and obese) confirmed a statistically significant difference (*p* <0.001), primarily driven by higher scores in the obese and overweight groups relative to normal-weight participants (Table S5, Fig. S2A). Conversely, no significant differences in model scores were observed among individuals with varying levels of alcohol consumption (light, moderate, or heavy; *p* = 0.248; Fig. S2B).

### PM scores closely align with most clinical variables commonly associated with increased risk for cardiovascular disease and heat maps focus on relevant structures

**Fig. 5:**
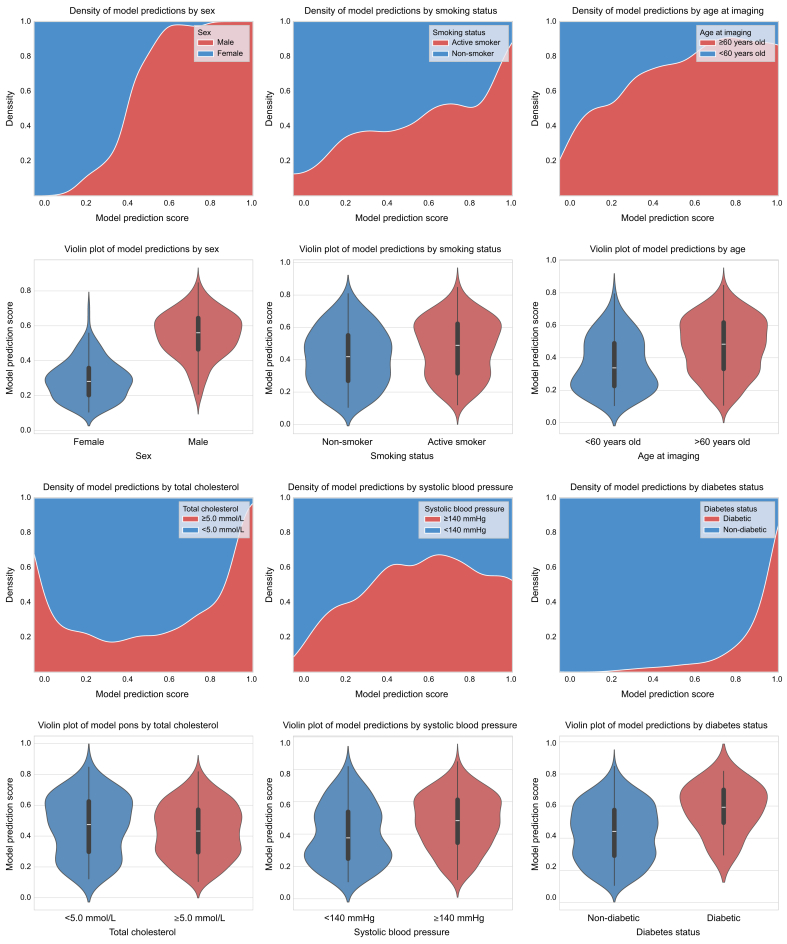
Density and violin plots of how participants were distributed based on their model prediction scores and variables used in SCORE2 to evaluate risk. Violin plot distributions (bottom) and corresponding probability density functions (top) of model predictions for various patient demographics and health indicators. Each plot illustrates the relationship between a specific patient characteristic (e.g., sex, age, smoking status, etc.) and the predicted score of the model, offering insights into the factors influencing its output. SCORE2, Systematic Coronary Risk Evaluation 2.

We observed the data based on density and violin plots (Fig. 5). We note that certain factors, such as positive diabetes status, male sex, positive smoking status, and low HDL cholesterol, were extremely strongly linked to higher PM scores. Notables, both extremely low and extremely high prediction scores had an over-representation of those with high total cholesterol values, indicating total cholesterol in isolation is not indicative of raised PM scores, and potentially, by extension, raised cardiovascular risk as well.

**Fig. 6:**
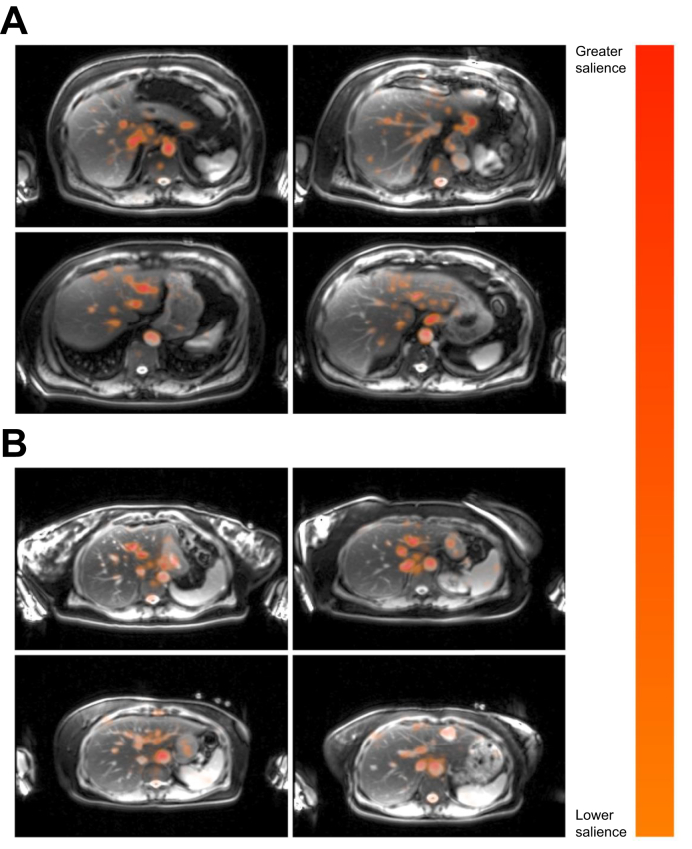
Heatmaps of demonstrating regions of high importance in model predictions. (A) True positives and (B) true negatives. The gradients, when multiplied with the pixel values of the image, provide a local approximation of the behavior of the model for specific input images. Areas with higher absolute gradient values indicate regions where small alterations in the input image strongly influence the prediction of the model. These heatmaps reveal the attention of the model to critical areas for predicting impending MACE or cardiovascular-related death. Consistently, focus is observed on the hepatic veins, inferior vena cava, and abdominal aorta, suggesting their significance in the decision-making process of the model. MACE, major adverse cardiac events.

We generated heatmaps visualizing the gradients of the input images after they were processed by the DL model (Fig. 6A,B). Upon observation of the MRI images generated, the model with high consistency focused in particular on three anatomical structures: the hepatic veins, inferior vena cava, and abdominal aorta.

### Proposed clinical utility

We propose that models such as ours can be integrated into current clinical practice alongside already established risk assessment tools, such as SCORE2.^5^^,^[36], [37], [38] However, clinical integration would require further prospective evaluation and validation in diverse healthcare settings. Such an approach could involve using the risk assessment provided by SCORE2 to initially decide the degree to which lifestyle and pharmacological intervention is necessary for a given patient. In patients whom a liver MRI is available, further risk assessment can be made with our model. Should the model determine that the patient is low risk, patient management can continue as suggested by SCORE2. In patients whose model prediction indicates elevated risk, a more aggressive treatment approach may be warranted. In this way, our model allows for more patients to be identified in whom more aggressive management of CVD risk factors is warranted (Fig. 1C). Future research should focus on conducting prospective studies to evaluate the real-time application of this model in clinical settings. Such studies are essential to assess its accuracy, implementation feasibility, and comparative effectiveness alongside existing tools, such as SCORE2. In addition, validation with external datasets, particularly from more diverse populations, will help ensure its robustness and generalizability.

## Discussion

The outcomes of our investigation further reinforce the potential of single-slice liver MRI images, and imaging techniques more broadly, when coupled with SSL techniques, to enhance the prediction of MACE and refine cardiovascular risk assessment. Our findings additionally add to the wealth of evidence pointing to the sizable capabilities of foundation models at achieving previously unobtainable levels of performance across a variety of downstream tasks.^35^^,^^41^ Several key points of our study warrant discussion, encompassing the performance of our PM, clinical implications, and the potential for risk stratification within specific subpopulations.

Our study demonstrates that applying SSL to single-slice liver MRI images can provide prognostically relevant insights into cardiovascular risk assessment. With a mean AUC of 0.70 derived through fivefold cross-validation, our model showed significant discriminatory capacity in predicting MACE and cardiovascular-related mortality, outperforming established methods, such as SCORE2. This aligns with our study objective of using liver MRI imaging to identify individuals at high risk of MACE.[31], [32], [33]

Survival analysis revealed a statistically significant difference in survival outcomes between individuals with PM scores above and below 0.5, underscoring the clinical relevance of our predictive model. This finding suggests that the PM score can serve as a valuable tool for identifying individuals at heightened risk of MACE, enabling timely interventions and personalized risk management strategies.^1^

Comparing the PM score with the SCORE2 model, we found similar diagnostic performance. The PM score independently predicted individuals at risk of MACE or cardiovascular-related death. With steeper ROC curves at lower specificity levels, the PM score may offer enhanced sensitivity. Notably, PM scores predicted MACE or death independently of well-established risk factors and current clinical metrics. Since SCORE2 is based on the UK Biobank dataset, the favorable performance of our model is noteworthy. In addition, the PM score provided better risk stratification when used alongside SCORE2, indicating its potential as a complementary tool for identifying patients needing more aggressive management.

The consistent focus of our model on the hepatic veins, inferior vena cava, and abdominal aorta in predicting MACE is strongly indicative of the role of these structures in the context of cardiovascular health. The prominence of the hepatic veins and vena cava likely reflects their role in venous return from the liver, indicating the metabolic and hemodynamic status of the liver. Liver function changes, such as fibrosis or steatosis, could affect hepatic venous outflow, which our model can capture. The significance of the abdominal aorta might stem from its role in systemic circulation and susceptibility to atherosclerosis.

Although our model exhibits promise in cardiovascular risk prediction, it is crucial to clarify its intended clinical application. We do not propose the widespread use of MRI as a general screening tool for cardiovascular risk in the population. Given the high cost and logistical challenges associated with MRI, our model is better suited for use in specific, high-risk patient groups or in cases where liver MRI data are already available, such as in individuals undergoing liver imaging for other purposes or those with underlying metabolic disorders.

Despite these promising findings, our study has limitations. The retrospective nature of the analysis and the reliance on a single-slice liver MRI image as a predictive feature could introduce bias or limit generalizability. We also faced constraints in incorporating detailed liver health metrics, particularly liver fat measurement data from the UK Biobank, because of recent changes in local data access policies. Although this information would have provided an important layer of analysis, distinguishing, for example, between participants with subclinical steatosis and those without any hepatic abnormalities, we were unfortunately unable to include it. Nevertheless, we did exclude participants with known liver disease (*i.e.* ICD-10 codes K70–K77) from our hold-out cohort to help ensure that the predictions of our model were not driven by overt hepatic pathology. This approach cannot fully account for mild or undiagnosed liver conditions, but does reduce the likelihood that advanced liver disease confounded our results. Future studies should consider including additional clinical and imaging data to enhance predictive accuracy. An additional factor is the lack of a fully independent external validation cohort, which raises questions regarding potential generalizability. In addition, while these results showcase the potential of recent advances in DL, the practicality of such risk assessment approaches in clinical practice could be largely limited by cost–benefit considerations. Our model is not intended to serve as a primary screening tool for CVD but rather as an adjunct for individuals already undergoing abdominal MRI for other indications. These may include imaging for liver disease assessment, cancer surveillance, or other gastrointestinal conditions. By leveraging existing imaging data without requiring additional dedicated scans, this method offers a cost-effective way to extract cardiovascular risk information from routine clinical workflows.

Furthermore, although we incorporated separate analyses examining PM score differences across BMI categories and alcohol consumption levels, these were not included in our main multivariable framework. We observed that participants classified as overweight or obese exhibited significantly higher PM scores compared with those with normal BMI, aligning with the well-established link between adiposity and cardiovascular risk. By contrast, alcohol consumption levels showed no significant association with PM scores. These findings underscore the importance of considering additional clinical and lifestyle factors in future analyses to more fully determine the independent predictive value of the imaging features of our model.

In conclusion, our study shows the potential of SSL applied to single-slice liver MRI images for cardiovascular risk assessment and MACE prediction. This approach could complement established risk assessment tools and guide personalized interventions, ultimately improving patient outcomes. However, we acknowledge that the integration of this model into clinical practice requires validation through prospective studies and external cohorts. Without such data, it is premature to recommend immediate adoption for routine clinical use. Nevertheless, our study is an important proof of concept and a strong motivation to conduct such extensive studies. Further research is needed to confirm these findings and improve model performance, facilitating the integration of such approaches into clinical practice.

## Abbreviations

AI, artificial intelligence; CVD, cardiovascular disease; DL, deep learning; ESC, European Society of Cardiology; HR, hazard ratio; ICD-10, International Classification of Diseases, 10th Revision; MACE, major adverse cardiac events; ML, machine learning; MoCo, Momentum Contrastive Learning; MRI, magnetic resonance imaging; NHS, National Health Service; PM, prediction model; ROC, receiver operating characteristic; SCORE2, Systematic Coronary Risk Evaluation 2; SSL, self-supervised learning; T1 MOLLI, T1 Mapping Modified Look-Locker Inversion Recovery; ViT, vision transformer; ViT-B, base Vit.

## Financial support

JNK is supported by the German Cancer Aid (DECADE, 70115166), the German Federal Ministry of Research, Technology and Space (PEARL, 01KD2104C; CAMINO, 01EO2101; TRANSFORM LIVER, 031L0312A; TANGERINE, 01KT2302 through ERA-NET Transcan; Come2Data, 16DKZ2044A; DEEP-HCC, 031L0315A; DECIPHER-M, 01KD2420A; NextBIG, 01ZU2402A), the German Academic Exchange Service (SECAI, 57616814), the German Federal Joint Committee (TransplantKI, 01VSF21048), the European Union’s Horizon Europe research and innovation programme (ODELIA, 101057091; GENIAL, 101096312), the European Research Council (ERC; NADIR, 101114631), the National Institutes of Health (EPICO, R01 CA263318) and the National Institute for Health and Care Research (NIHR) Leeds Biomedical Research Centre (grant number NIHR203331). The views expressed are those of the author(s) and not necessarily those of the NHS, the NIHR or the Department of Health and Social Care. This work was funded by the European Union. Views and opinions expressed are however those of the author(s) only and do not necessarily reflect those of the European Union. Neither the European Union nor the granting authority can be held responsible for them.

## Authors’ contributions

Designed the study: GPV, JNK. Performed the experiments: GPV and T Lenz. Analyzed the data: GPV and JNK. Collated the relevant tabular data and its derived metrics: DC and GPV. Performed statistical analyses: GPV. Provided clinical expertise and contributed to the interpretation of the results: all authors. Wrote the manuscript with assistance from T Lenz: GPV. Provided feedback on the manuscript and collectively made the decision to submit for publication: all authors.

## Data availability statement

Data can be accessed through an application to the UK Biobank upon approval (https://www.ukbiobank.ac.uk/).

## Conflicts of interest

JNK declares consulting services for Owkin, DoMore Diagnostics, and Panakeia; furthermore, he holds shares in StratifAI GmbH and has received honoraria for lectures from Bayer, Eisai, MSD, BMS, Roche, Pfizer, and Fresenius. DT has received honoraria for lectures from Bayer and holds shares in StratifAI GmbH. T. Luedde has received honoraria for lectures from AbbVie, AstraZeneca, BMS, Gilead, MSD, and Roche.

Please refer to the accompanying ICMJE disclosure forms for further details.
