# Supplementary material for: Deep learning can predict cardiovascular events from liver imaging
Source: JHEP Rep. 2025 Apr 22;7(8):101427. doi: 10.1016/j.jhepr.2025.101427 (PMC12260415; doi:10.1016/j.jhepr.2025.101427)

Deep learning can predict cardiovascular events from liver imaging

Gregory Patrick Veldhuizen, Tim Lenz, Didem Cifci, Marko van Treeck, Jan Clusmann, Yazhou Chen, Carolin V. Schneider, Tom Luedde, Peter W. de Leeuw, Ali El-Armouche, Daniel Truhn, Jakob Nikolas Kather

Table of contents

Table S1…………………………………………………………………………….……..2

Table S2…………………………………………………………………………….……..3

Table S3…………………………………………………………………………….……..3

Table S4…………………………………………………………………………….……..4

Table S5…………………………………………………………………………….……..5

Table S6…………………………………………………………………………….……..6

Fig. S1………………………………………………………………………………………10

Fig. S2………………………………………………………………………………………11

Table S1. ICD10-codes used to define major adverse cardiac events (MACE).

**Fatal cardiovascular disease-cause specific mortality due to any of the following:**	
**Endpoints included**	**ICD10-codes**
Hypertensive disease	I10-16
Ischemic heart disease	I20-25
Arrhythmias, heart failure	I46-52
Cerebrovascular disease	I60-69
Atherosclerosis/AAA	I70-73
Sudden death and death within 24h of symptom onset	R96.0-96.1
**Endpoints excluded from the above endpoint:**	**ICD10-codes**
Myocarditis, unspecified	I51.4
Subarachnoid hemorrhage	I60
Subdural hemorrhage	I62
Cerebral aneurysm	I67.1
Cerebral arteritis	I68.2
Moyamoya	I67.5
**Non-fatal cardiovascular disease:**	
**Endpoints included**	**ICD10-codes**
Non-fatal myocardial infarction	I21-123
Non-fatal stroke	I60-69
**Excluded from the non-fatal stroke endpoint:**	**ICD10-codes**
Subarachnoid hemorrhage	I60
Subdural hemorrhage	I62
Cerebral aneurysm	I67.1
Cerebral arteritis	I68.2
Moyamoya	I67.5

Table S2. Pretraining hyperparameters

**Hyperparameter**	**Value**
learning rate	1e-4
weight decay	0.1
epochs	2000
warmup epochs	30
moco temperature	0.2
batch size	1024
optimizer	AdamW
architecture	vit_conv_base

Table S3. Classifier hyperparameters

**Hyperparameter**	**Value**
learning rate	3e-4
batch size	512
early stopping	8
max epochs	420

Table S4. Patient Characteristics

Variable	All Mean (n=43,698)	All SD	Male Mean (n=19,926)	Male SD	Female Mean (n=23,772)	Female SD
Age (years)	64.4	7.8	65.2	7.9	63.7	7.7
Weight (kg)	76.4	15.3	83.9	13.6	69.2	13.4
Height (cm)	170.2	9.4	177.1	6.6	163.5	6.4
BMI (kg/m^2)	26.6	4.5	27.1	4	26.2	4.8
HDL (mmol/L)	1.5	0.4	1.3	0.3	1.6	0.4
LDL (mmol/L)	3.6	0.8	3.6	0.8	3.6	0.8
Systolic BP (mmHg)	139.8	19	142.6	17.6	137.1	19.8
Diastolic BP (mmHg)	78.9	10.1	80.6	9.9	77.3	10.1
Heart Rate (bpm)	61.8	10.7	60.8	11	62.7	10.3

Table S5. ANOVA with post-hoc Tukey HSD Test for Model Scores and BMI categories.

**BMI Categories Compared**		**Mean Difference**	**Adjusted P-Value**
normal	obese	0.1503	<0.001
normal	overweight	0.1119	<0.001
normal	underweight	-0.0895	0.8738
obese	overweight	-0.0384	0.2699
obese	underweight	-0.2398	0.1837
overweight	underweight	-0.2014	0.3225

Table S6: TRIPOD-AI Table.

Topic	Item	Description	Reported on Page
**TITLE**	1	Identify the study as developing or evaluating the performance of a multivariable prediction model, the target population, and the outcome to be predicted	Title Page
**INTRODUCTION**	3a	Explain the healthcare context and rationale for developing or evaluating the prediction model, including references to existing models	Introduction
	3b	Describe the target population and the intended purpose of the prediction model in the context of the care pathway, including its intended users	Introduction
	3c	Describe any known health inequalities between sociodemographic groups	N/A
	4	Specify the study objectives, including whether the study describes the development or validation of a prediction model (or both)	Introduction
**METHODS**	5a	Describe the sources of data separately for the development and evaluation datasets, the rationale for using these data, and representativeness of the data	Methods
	5b	Specify the dates of the collected participant data, including start and end of participant accrual; and, if applicable, end of follow-up	Methods
	6a	Specify key elements of the study setting including the number and location of centers	Methods
	6b	Describe the eligibility criteria for study participants	Methods
	6c	Give details of any treatments received, and how they were handled during model development or evaluation, if relevant	Methods
	7	Describe any data pre-processing and quality checking, including whether this was similar across relevant sociodemographic groups	Methods
	8a	Clearly define the outcome that is being predicted and the time horizon, including how and when assessed, the rationale for choosing this outcome, and whether the method of outcome assessment is consistent across sociodemographic groups	Methods
	8b	If outcome assessment requires subjective interpretation, describe the qualifications and demographic characteristics of the outcome assessors	N/A
	8c	Report any actions to blind assessment of the outcome to be predicted	N/A

	9a	Describe the choice of initial predictors and any pre-selection of predictors before model building	Methods
	9b	Clearly define all predictors, including how and when they were measured (and any actions to blind assessment of predictors for the outcome and other predictors)	Methods
	9c	If predictor measurement requires subjective interpretation, describe the qualifications and demographic characteristics of the predictor assessors	N/A
	10	Explain how the study size was arrived at (separately for development and evaluation), and justify that the study size was sufficient to answer the research question. Include details of any sample size calculation	Methods
	11	Describe how missing data were handled. Provide reasons for omitting any data	Methods
	12a	Describe how the data were used (e.g., for development and evaluation of model performance) in the analysis, including whether the data were partitioned, considering any sample size requirements	Methods
	12b	Describe how predictors were handled in the analyses (functional form, rescaling, transformation, or any standardisation)	Methods
	12c	Specify the type of model, rationale, all model-building steps, including any hyperparameter tuning, and method for internal validation	Methods
	12d	Describe if and how any heterogeneity in estimates of model parameter values and model performance was handled and quantified across clusters	N/A
	12e	Specify all measures and plots used (and their rationale) to evaluate model performance and, if relevant, to compare multiple models	Methods
	12f	Describe any model updating (e.g., recalibration) arising from the model evaluation, either overall or for particular sociodemographic groups or settings	N/A
	12g	For model evaluation, describe how the model predictions were calculated (e.g., formula, code, object, application programming interface)	Methods
	13	If class imbalance methods were used, state why and how this was done, and any subsequent methods to recalibrate the model or the model predictions	Methods
	14	Describe any approaches that were used to address model fairness and their rationale	N/A

	15	Specify the output of the prediction model. Provide details and rationale for any classification and how the thresholds were identified	Methods
	16	Identify any differences between the development and evaluation data in healthcare setting, eligibility criteria, outcome, and predictors	Methods
	17	Name the institutional research board or ethics committee that approved the study and describe the participant-informed consent or the ethics committee waiver of informed consent	Methods
**OPEN SCIENCE**	18a	Give the source of funding and the role of the funders for the present study	Acknowledgements
	18b	Declare any conflicts of interest and financial disclosures for all authors	Acknowledgements
	18c	Indicate where the study protocol can be accessed or state that a protocol was not prepared	N/A
	18d	Provide registration information for the study, including register name and registration number, or state that the study was not registered	N/A
	18e	Provide details of the availability of the study data	Acknowledgements
	18f	Provide details of the availability of the analytical code	Acknowledgements
**PATIENT & PUBLIC INVOLVEMENT**	19	Provide details of any patient and public involvement during the design, conduct, reporting, interpretation, or dissemination of the study or state no involvement	N/A
**RESULTS**	20a	Describe the flow of participants through the study, including the number of participants with and without the outcome and, if applicable, a summary of the follow-up time. A diagram may be helpful	Results
	20b	Report the characteristics overall and, where applicable, for each data source or setting, including the key dates, key predictors (including demographics), treatments received, sample size, number of outcome events, follow-up time, and amount of missing data. A table may be helpful. Report any differences across key demographic groups	Results
	20c	For model evaluation, show a comparison with the development data of the distribution of important predictors (demographics, predictors, and outcome)	N/A
	21	Specify the number of participants and outcome events in each analysis (e.g., for model development, hyperparameter tuning, model evaluation)	Results
	22	Provide details of the full prediction model (e.g., formula, code, object, application programming interface) to allow predictions in	N/A

		new individuals and to enable third-party evaluation and implementation, including any restrictions to access or re-use	
	23a	Report model performance estimates with confidence intervals, including for any key subgroups. Consider plots to aid presentation	Results
	23b	If examined, report results of any heterogeneity in model performance across clusters	N/A
	24	Report the results from any model updating, including the updated model and subsequent performance	N/A
**DISCUSSION**	25	Give an overall interpretation of the main results, including issues of fairness in the context of the objectives and previous studies	Discussion
	26	Discuss any limitations of the study and their effects on any biases, statistical uncertainty, and generalizability	Discussion
	27a	Describe how poor quality or unavailable input data should be assessed and handled when implementing the prediction model	Discussion
	27b	Specify whether users will be required to interact in the handling of the input data or use of the model, and what level of expertise is required of users	Discussion
	27c	Discuss any next steps for future research, with a specific view to applicability and generalizability of the model	Discussion

Fig. S1. CONSORT chart

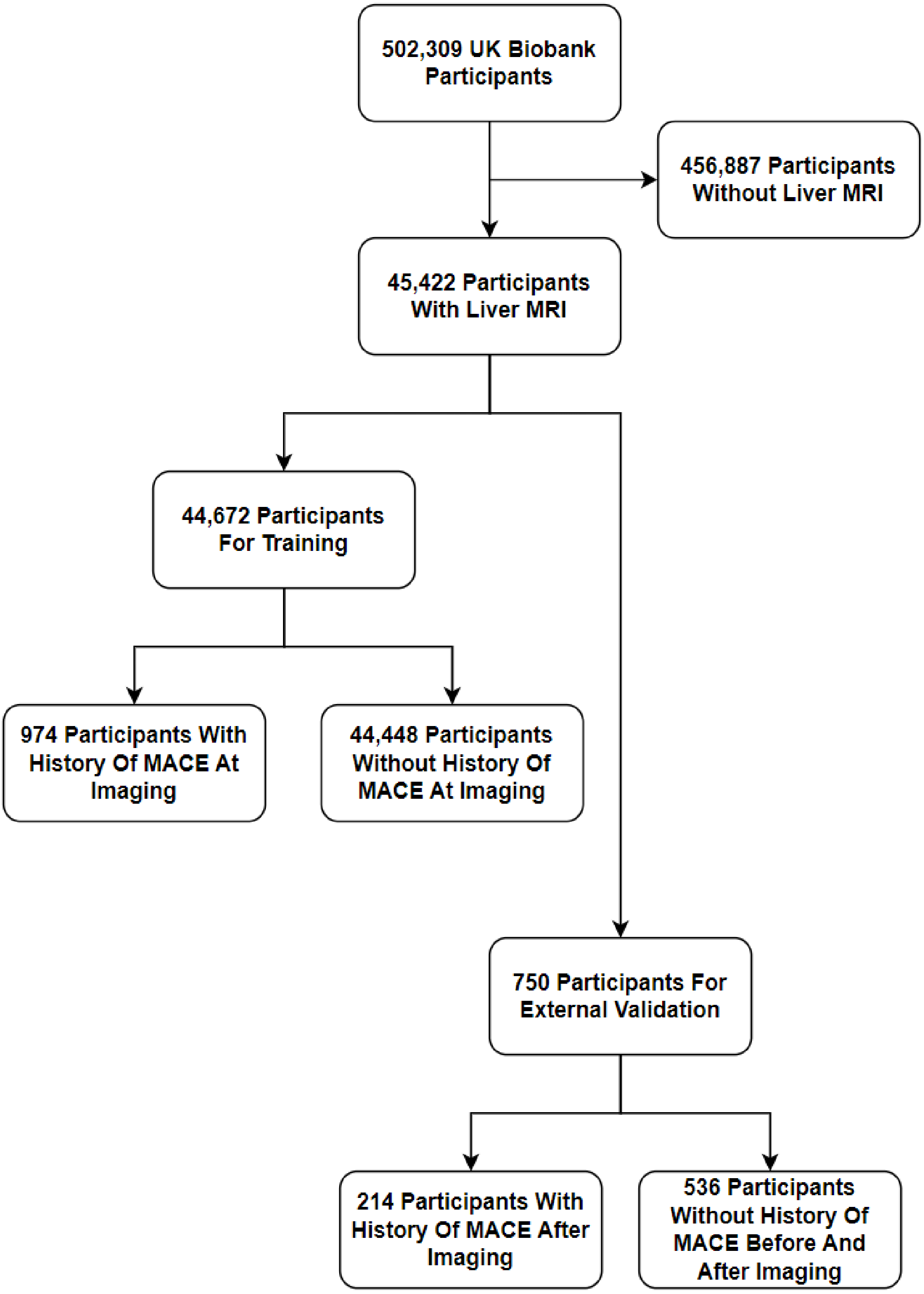

Fig. S2. Panel A: PM Score Box Plots for BMI categories. Panel B: PM Score Box Plots for Alcohol Status categories.

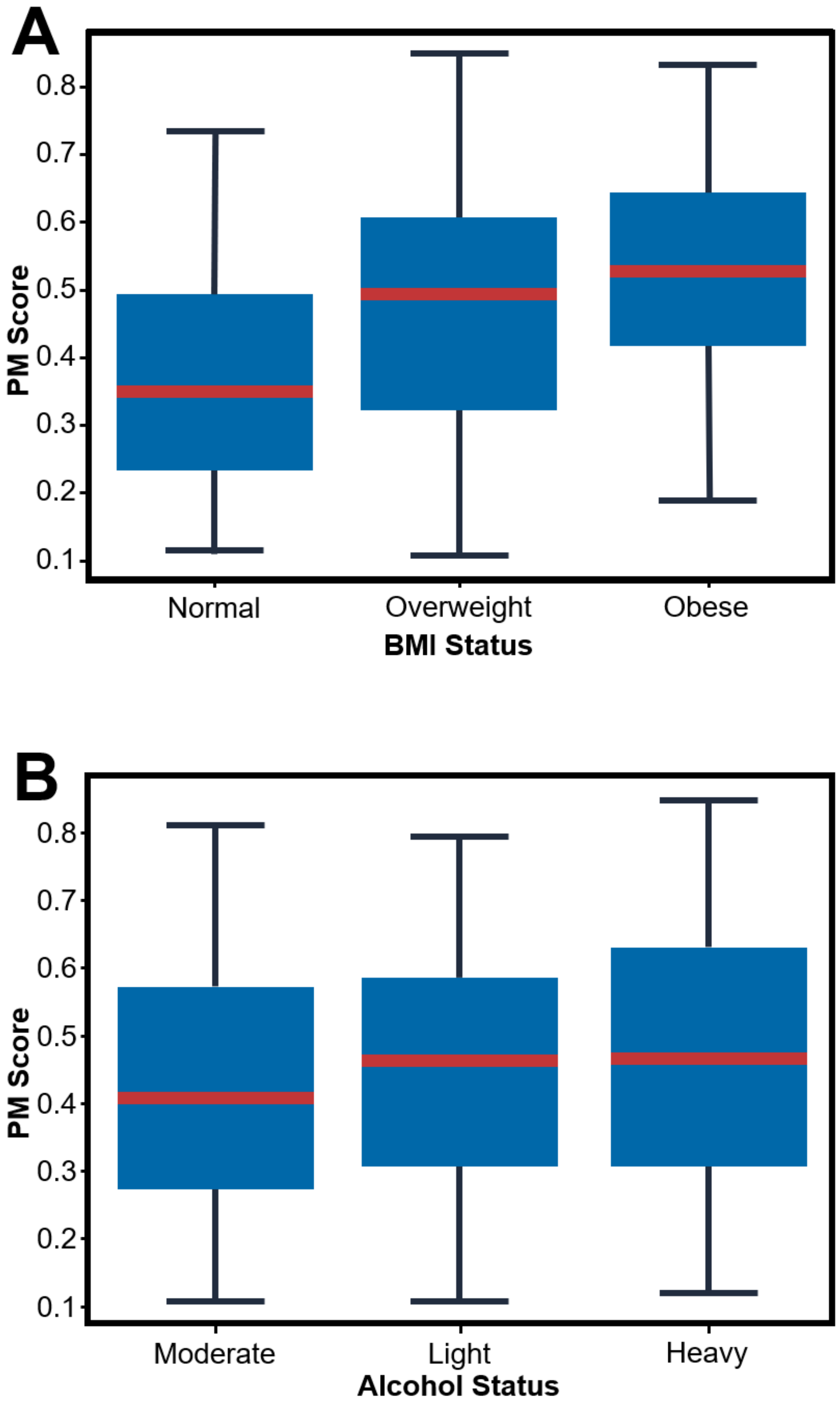

Supplement: Multimedia component 1 [file mmc1.pdf]
