# Supplementary material for: Deep learning can predict cardiovascular events from liver imaging
Source: JHEP Rep. 2025 Apr 22;7(8):101427. doi: 10.1016/j.jhepr.2025.101427 (PMC12260415; doi:10.1016/j.jhepr.2025.101427)
Supplement: Multimedia component 2 [file mmc2.pdf]

JOURNAL
OF HEPATOLOGY

Journal of Hepatology

CTAT methods

Tables for a "Complete, Transparent, Accurate and Timely account" (CTAT) are now mandatory for all revised submissions. The aim is to enhance the reproducibility of methods.

- Only include the parts relevant to your study
- Refer to the CTAT in the main text as 'Supplementary CTAT Table'
- Do not add subheadings
- Add as many rows as needed to include all information
- Only include one item per row

If the CTAT form is not relevant to your study, please outline the reasons why:

We don't believe CTAT is relevant to our study and our study is retrospective EMR (electronic medical record) based study.

1.1 Antibodies: (not applicable)

Name	Citation	Supplier	Cat no.	Clone no.

1.2 Cell lines: (not applicable)

Name	Citation	Supplier	Cat no.	Passage no.	Authentication test method

1.3 Organisms: (not applicable)

Name	Citation	Supplier	Strain	Sex	Age	Overall n number

1.4 Sequence based reagents: (not applicable)

Name	Sequence	Supplier

1.5 Biological samples: (not applicable)

Description	Source	Identifier

1.6 Deposited data: Research data is confidential.

Name of repository	Identifier	Link

1.7 Software

Software name	Manufacturer	Version

1.8 Other (e.g. drugs, proteins, vectors etc.) : (not applicable)

1.9 Please provide the details of the corresponding methods author for the manuscript:

Professor Jakob Nikolas Kather, MD, MSc email: jakob-nikolas.kather@alumni.dkfz.de

2.0 Please confirm for randomised controlled trials all versions of the clinical protocol are included in the submission. These will be published online as supplementary information.

(not applicable)
